# Antioxidant and Hepatoprotective Activity of a New Tablets Formulation from* Tamarindus indica* L.

**DOI:** 10.1155/2016/3918219

**Published:** 2016-04-07

**Authors:** Jesús Rafael Rodriguez Amado, Ariadna Lafourcade Prada, Julio Cesar Escalona Arranz, Renato Pérez Rosés, Humberto Morris Quevedo, Hady Keita, Edgar Puente Zapata, Caio Pinho Fernandes, José Carlos Tavares Carvalho

**Affiliations:** ^1^Laboratório de Pesquisa em Fármacos, Universidade Federal do Amapá, 68906-970 Macapá, AP, Brazil; ^2^Departamento de Farmacia, Universidad de Oriente, 90500 Santiago de Cuba, Cuba; ^3^Centro de Toxicología y Biomedicina, Universidad Médica de Santiago de Cuba, 90500 Santiago de Cuba, Cuba; ^4^Laboratório de Nanobiotecnologia Fitofarmacêutica, Universidade Federal do Amapá, 68906-970 Macapá, AP, Brazil

## Abstract

Hepatotoxic chemicals damage liver cells primarily by producing reactive oxygen species. The decoction of the leaves of* Tamarindus indica* L. is used for liver disorders. In this work we evaluated the hepatoprotective activity of a tablet formulation of this plant. Thirty-five Sprague Dawley rats were randomly divided into five groups (*n* = 7). First group (I) is control group, fed with standard diet. Groups II to V (hepatotoxic groups) were subjected to a subcutaneous injection of CCl_4_ (0.5 mL/kg). Group II was negative control, fed with standard diet; group III was subjected to administration of Silymarin 150 mg/kg and groups IV and V were treated with tablets in dose of 100 mg/kg and 200 mg/kg, respectively. Lipid peroxidation and the activity of superoxide dismutase, catalase, and reduced glutathione were evaluated. Serum levels of alanine aminotransferase, aspartate aminotransferase, gamma-glutamine transferase, alkaline phosphatase, and a lipid profile were evaluated too. The tablets inhibit lipid peroxidation. The redox balance (SOD-CAT-GSH) remains normal in the experimental groups treated with tablets. The liver function using dose of 200 mg/kg of tablets was better than the other experimental groups. These results justify, scientifically, the ethnobotanical use of the leaves of* Tamarindus indica* L.

## 1. Introduction

As it is known, the production of reactive oxygen species (ROS) by cells leads to oxidative stress and macromolecular damage, contributing to the pathogenesis of many diseases. Liver damage is very common because the liver is a key organ in the detoxification process. Hepatotoxic chemicals damage liver cells primarily, by producing ROS, some of which form covalent bonds with the lipid tissue. Due to excessive exposure to hazardous chemicals, sometimes the free radicals generated are so high that they overpower the natural defense system, leading to hepatic damage [1].

The peroxidation of membrane lipids results in the loss of membrane structure and integrity. This results in elevated serum levels of enzymes, especially *γ*-glutamyl transpeptidase, a membrane bound enzyme in serum [2]. Acute poisoning with carbon tetrachloride (CCl_4_) causes a high lipid accumulation. After that, a necrosis of the tissue is presented. Some chemicals produce a very specific type of damage; others notably ethanol produce sequential types of damage or combination of damages. Hepatitis, a common disorder of varying severity, can lead to cirrhosis, liver failure, and death. If acute liver disorders are not promptly treated the damage will go to chronic forms characterized by continuing hepatocellular necrosis and inflammation, usually with fibrosis, which tends to progress to cirrhosis and liver failure [3]. In this context, therapeutic alternatives are limited. For this reason, there is a great need to find new drugs for the treatment of these pathologies.

*Tamarindus indica* L. (TIL) or tamarind as is commonly known belongs to the Fabaceae family, Caesalpiniaceae subfamily; it is a tropical tree, native of Africa and Southern Asia. This plant is widespread in the Amazonia and the Caribbean. The decoction of its leaves constitutes a useful remedy for hepatitis, jaundice, and gallbladder disorders treatment in the Caribbean area and the Amazon [4]. Tamarind leaves contain fatty acids, heavy alcohols, proteins, and essential amino acids and carbohydrates [5]. Important minerals for cell redox balance as zinc, manganese, copper, nickel, and selenium are reported for the leaves of this species, too [6].

Around 20 volatile oils are reported in the leaves of this species [7]. Polyphenols (i.e., ferulic and caffeic acids) and flavonoids (orientin, isoorientin, vitexin, and isovitexin) are reported as mainly responsible for the strong antioxidant activity [8], hepatoprotective activity [9], and antimicrobial [6] of the leaves extracts of this plant. Other metabolites reported for tamarind leaves are triterpenes (lupeol and lupanone), fatty acids (palmitic and oleic acid), and others as tartaric and citric acids and vitamins (A, C, and E) [5].

The majority of the tamarind constituents are antioxidants; for this reason it has been used for centuries as hepatoprotective. In this way, it has been reported as strong antioxidant activity of the polyphenols and flavonoids, mediated by the inhibition of ROS mechanism formation [1, 10]. The antioxidant effects of essential oils are strongly dependent on the content of phenolic [11]. Ferulic acid protects from CCl_4_ induced acute liver injury through reduction of oxidative damage and inflammatory signaling pathways [12]. Copper, nickel, manganese, and iron are involved in the first line of the endogenous antioxidant cell defense system [3] and selenium in association with vitamin E plays an important role in the protection of the membrane lipid content [13].

The aim of this work was to evaluate the antioxidant and hepatoprotective activity of a new tablet formulation obtained from the standardized soft extract of* Tamarindus indica* L. leaves.

## 2. Materials and Methods

### 2.1. Plant Material

Tamarind leaves were collected (November 2014) from a tamarind population in Santiago de Cuba, Cuba (located 20°2′38.9′′N and 075°45′25.8′′W). A voucher specimen (registered as 052216) was deposited at the herbarium of the Biology Department, University of Oriente, Cuba. Collected leaves were sun-dried (residual humidity below 10% by the stove method), milled (MLK, Russia), and passed across 5 mm of mesh light sieve.

### 2.2. Tablets Preparation

Tablets of* Tamarindus indica* L. were obtained and optimized by using the wet granulation method [14]. The standardized soft extract from* Tamarindus indica* L. leaves was used as active ingredient [15]. Tablets contain excipients like microcrystalline cellulose, lactose monohydrate, polyvinylpyrrolidone, croscarmellose sodium, colloidal silicon dioxide, and magnesium stearate, all of which are approved by the FDA [16].

### 2.3. Animals

Thirty-five adult female Sprague Dawley rats, weighing 150–200 g each, were obtained from the National Center for the Production of Laboratory Animals (CENPALAB), Havana City, Cuba. Rats were housed in Makrolon cages (seven per cages) under normal laboratory conditions. They were fed with standard diet CMO-1000 (CENPALAB, Havana) for one week as an adaptation period. Water was provided to rats from an inverted bottle supported on the top of the cage. Food and water were provided* ad libitum*.

All the experiments were carried out in agreement with the Good Laboratory Practices, taking into account the ethical considerations settled down, in the Guide for the Handling of the Laboratory Animal of the International Council for Laboratory Animals Sciences [17], according to the Ethical Committee of the Toxicology and Biomedicine Center (TOXIMED), Medical University of Santiago de Cuba, Cuba.

### 2.4. Experimental Groups

Thirty-five rats were randomly divided into five groups (*n* = 7). The first group (I) was normal untreated group, fed with standard diet and distilled water all the time. Hepatotoxic groups (groups II to V) were subjected to subcutaneous injection of CCl_4_ in a single dose of 0.5 mL/kg, mixed with an equal volume of soya oil, on the 2nd day of experiment [18]. The second group (II) was the negative control, fed with standard diet and distilled water, without any treatment. The third group (III) was treated with Silymarin 150 mg/kg (Cosmos, México) as standard hepatoprotective drug and groups IV and V were treated with TIL tablets at doses 100 mg/kg and 200 mg/kg, respectively. Rats were weighted at 0, 4, and 7 days.

### 2.5. Food and Water Intake

The intake of water and food was measured daily. Glass transparent flasks of 250 mL were utilized. They were tared, so each division corresponds to 10 mL. The flasks were daily completed with water. They were plugged utilizing an adjustable closing of PVC. This permitted a daily control of the water consumed. A quantity of 175 g of food was supplied initially, and it was placed in topside of the box. Daily, the remnant food was weighed and the initial mass was completed again. The measurements were made until the seventh day of experiment.

### 2.6. Biochemical Assays

#### 2.6.1. Blood and Tissue Collection

Experiment was concluded at 7 days. Rats were starved for 12 h and then sacrificed under light ether anesthesia. The blood was obtained from all animals by puncturing retroorbital plexus. The blood samples were collected into clean dry centrifuge tubes stored at room temperature for 10 minutes and then at 4°C for one hour and centrifuged at 4000 rpm for 15 minutes to separate serum. Serum was transferred into dry clean tubes and preserved at −20°C until being assayed.

The rat livers were separated and washed in the ice-cold saline solution (1–4°C), dried with filter paper, and weighed immediately. The homogenate (prepared in 0.1 M Tris-HCl buffer at pH 7.2) was centrifuged at 15000 rpm for 5 minutes and supernatant was used for the assay [19].

*Antioxidant Activity*. The antioxidant activity of the tablets was evaluated on hepatic tissue samples. The lipid peroxidation (LP) was evaluated using the malondialdehyde (MDA) concentration, as an indirect measure [20]. The Han et al. [21] method was used to determine MDA, based on its reaction with thiobarbituric acid to form a pink complex with maximum absorption at 535 nm. The superoxide dismutase (SOD) enzymatic activity was determined using the Superoxide Dismutase Assay Kit, from Cayman Chemical Company (USA). Catalase activity was determined using the commercial kit CAT-240 (Applied Bioanalytical Labs, USA). Reduced glutathione (GSH) concentration was measured using the Glutathione Assay Kit (Sigma-Aldrich®, USA).

*Hepatoprotective Activity*. Serum levels of alanine aminotransferase (ALT), aspartate aminotransferase (AST), gamma-glutamine transferase (GGT), alkaline phosphatase (ALP), and total bilirubin (TB) were determined using commercially available kits (Spinreact, Spain), according to the manufacturer's instructions. Total protein (TP) was evaluated using the Biuret method depicted in commercial kit (HELFA, Diagnostics, Cuba).

Serum total cholesterol, triglycerides (TG), and high-density lipoproteins cholesterol (HDL-c) were determined using the methods described by of Allain et al. [22], respectively. The determination of low-density lipoproteins cholesterol (LDL-c) and very low-density lipoproteins (VLDL-c) was performed according to the methods described by Lee and Nieman [23].

### 2.7. Statistical Analysis

Statgraphics plus (version 5.0.1 for Windows, MA, USA) was used to carry out the statistical analysis. The one-way analysis of variance (ANOVA) and Tukey HSD test were carried out to compare the groups that were statistically different. Values of *p* < 0.05 were considered significant.

## 3. Results 

### 3.1. Corporal Weight and Intake of Water and Food

**Figure 1:**
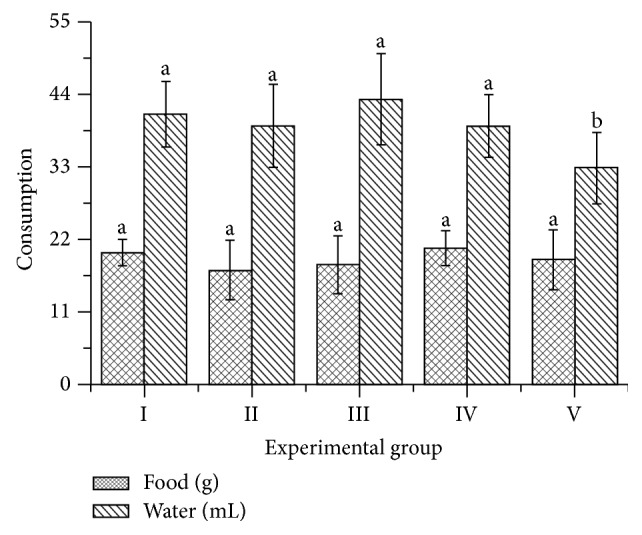
Intake of water and foods for the animals during the experiment. I: control group; II: CCl_4_ induced hepatotoxicity group; III: Silymarin treated group; IV and V:* Tamarindus indica* L. tablets treated groups at doses 100 mg/kg and 200 mg/kg, respectively. Different letters on columns indicate significant statistical differences (Tukey HSD, *p* < 0.05).

Figure 1 shows the daily intake of food and water of all experimental groups, while Table 1 does it for the corporal weight and liver weight behavior. The intake of water was the same for groups from I to IV, but statistically higher than those measured for group V (*F* = 3.20; *p* = 0.0265). On the other hand, the food intake was statistically the same for all groups (*F* = 0.58; *p* = 0.5480). Relating these results to the corporal weight gain (Table 1) it is a fact that, independently of treatment group, the animals do not modify their habits of nutrition and hydration. This has a direct impact on their corporal weight increment with the exception of group II, in which significant statistical differences were found. In general, there is an inverse relationship between corporal weight increments and liver weight as should happen in hepatic disorders [24]. According to the anatomical variable (liver weight/corporal weight relationship), the three treated groups (III, IV, and V) showed a preserved hepatic function, in a clear opposition to the liver damage induced by the carbon tetrachloride injection.

### 3.2. Antioxidant and Hepatoprotective Activity

Table 2 shows the different antioxidant variables having into account all the five experimental groups. In all cases, the treatment with Silymarin or* Tamarindus indica* L. tablets activated the antioxidant defense system in a similar way and at normal levels (compared to group I), with the only exception of MDA. Data do not reveal clear differences between the two doses of tamarind tablets used.  Table 3 shows the behavior of the hepatic function of the rats in the experimental groups. Increments in the activity of enzymes ALT, AST, ALP, and GGT in the tetrachloride group were observed. The values of the activity of these enzymes were bigger and statistically different from the control group (*P* < 0.05). The ALT activity observed for groups III, IV, and V decreased their levels at the same rank as the control group (I) without statistical differences between them. For the other enzymes (AST, ALP, and GGT) the activity level decreased regarding group II but did not reach the normal values of group I. In general, the group treated with 200 mg/kg of tamarind tablets exhibit the better results, in some cases better than Silymarin group.

Table 4 shows effect of* Tamarindus indica* L. tablets on serum lipid profile of Sprague Dawley rats. Treatment with CCl_4_ produced significant elevations in serum triglyceride concentrations (*p* < 0.05). However, the group treated with* Tamarindus indica* L. tablets at both doses (100 and 200 mg/kg) had the lowest triglyceride levels. Cholesterol levels had a similar pattern to that of the triglycerides. Notably, the CCl_4_ treatment increased the LDL-c concentration (group II). The treatment with* Tamarindus indica* L. tablets significantly reduced the LDL-c levels, particularly at the dose of 200 mg/kg (*p* < 0.05). Supplementation with* Tamarindus indica* L. tablets led to decreasing in LDL-c levels higher than the Silymarin treated group. As expected, the highest concentrations of VLDL-c were found in CCL_4_ treated animals. The administration of *Tamarindus indica *L. tablets especially at 200 mg/kg led to statistically significant decrease of VLDL-c values (*p* < 0.05) compared to group II (CCl_4_), but its effectiveness was lower than in the Silymarin group.

## 4. Discussion

The weight gain observed in groups III, IV, and V indicates that despite the presence of the CCl_4_, the treatment with tamarind tablets was able to maintain the organic functionality of the biomodels. The Silymarin and tablets administered at both doses protect the biosynthetic function of animals. In contrast, in animals in Group II even when their food and water intake was similar to the rest of the experimental groups, their body weight decreased after the fourth day. This suggests that there was a decrease in biosynthetic function caused by the toxic action of CCl_4_.

The hepatotoxic effects of the CCl_4_ are associated with trichloromethyl free radical [25]. Thus, the free radicals bind covalently to the macromolecules inducing peroxidative degradation of membrane lipids in the endoplasmic reticulum, which is rich in polyunsaturated fatty acids. This leads to the formation of lipid peroxides and the degradation of membranes. The latest is the principal causes of hepatotoxicity of CCl_4_ [26].

The polyphenol and flavonoids content in TIL extracts are responsible for the antioxidant [8] and hepatoprotective [9] activity of this drug. On the other hand, the elemental contents, mainly copper, nickel, manganese, and iron, are cofactors of the four isoenzymes of the SOD. These enzymes in joint action with glutathione peroxidase and glutathione reductase play an important role as part of the antioxidant defense system [24]. The last one is selenium-dependent. Selenium plays an important role as antioxidant [27]. This microelement combined with vitamin E plays important role in the protection of the membrane lipid content [13].

Lipid peroxidation plays an important role in decompensated liver function [28, 29]. Propagation reactions associated with lipid peroxidation produce MDA, the indicator most commonly used to assess lipid peroxidation [2, 30]. In this research, a high concentration of MDA in CCl_4_ treated group was observed. Tamarind tablets in both dose levels significantly decreased the production of MDA in the liver of experimental animals. This is an evidence of an inhibitory activity of lipid peroxidation, similar to that produced by Silymarin 150 mg/kg. The literature reports that several groups of metabolites present in the phytocomplex of the tamarind soft extract contribute to antioxidant activity by different ways. Thus, antioxidants act together in their effects [31]. This is the case of exogenous nonenzymatic antioxidants such as vitamins C and E for keeping the integrity of the hepatocyte membrane [32] and the endogenous enzymatic system (superoxide dismutase-catalase-glutathione) inside the hepatocytes [2, 33].

The results suggest that not only polyphenols and flavonoids are responsible for the observed antioxidant effect. In this way, the presence of the elements like zinc, copper, manganese, iron, and selenium; tartaric, citric, malic [34], palmitic, and oleic acid [35, 36]; and vitamins of the B complex and triterpenes as lupanone and lupeol [37] is important, all of which have an important antioxidant activity. Probably, a synergy may occur with the activity of other constituents present in the soft extract, fact that is reported in literature [31]. Synergy would be a plausible explanation for the good antioxidant activity observed in this work.

In plant extracts, both the antioxidant effect and the hepatoprotective activity cannot be attributed to a particular substance. Numerous reports suggest that the “synergy” of all metabolites present in the active principle has a powerful antioxidant and hepatoprotective activity. This action is produced throughout various mechanisms [38, 39]. In this regard, the potential of* Tamarindus indica* L. tablets is very high. The synergy was reported in the hepatoprotective activity of some substances present in natural extracts, among them, vitamin C and *α*-tocopherol [40, 41]; linalool and polyphenols [11]; *γ*-terpinene and p-cymene [42]; and *α*-tocopherol and *β*-sitosterol [40]. Other synergistic effects are reported too for ferulic acid and caffeic acid with their phenyl esters [43] and selenium with vitamin E [13]. All of these metabolites are present in the soft extract used as active principle in tamarind tablets.

Carbon tetrachloride increased dramatically the serum triglyceride concentrations. The pathogenesis of fatty liver in CCl_4_ intoxication is the result of an imbalance between hepatic fatty acid flow and triglycerides synthesis and excretion. One of the earliest manifestations of CCl_4_ induced liver damage is the accumulation of fat. Characteristic gene expression profiles may be associated with the disruption of lipid metabolism induced by CCl_4_ treatment [37].

Treatment with* Tamarindus indica* L. tablets at both doses led to a significant reduction of triglyceride and cholesterol levels in rats. These results, at least partially, could reflect the VLDL-c pattern found in our study, lower than the animals treated with CCl_4_ and similar to the group treated with Silymarin. The VLDL-c is involved in the transport of endogenous triglycerides from liver to peripheral tissues and is the precursor of LDL-c, through the intermediate density lipoproteins [24]. The LDL-c transports cholesterol to diverse tissues and in conditions in which their level increased above normal they represent a risk for atherosclerosis development. In our work, the LDL-c was restored to values of the control group. Another interesting fact is the increasing of HDL-c in the animals treated with* Tamarindus indica* L. tablets with respect to animals treated with CCl_4_ alone. HDL-c plays a crucial role in the cholesterol reverse transport from peripheral tissues to liver for its excretion [24]. In general, these findings suggest an improvement in the lipid profile as the result of the activity of tamarind tablets administration to CCl_4_ intoxicated rats. Chan et al. [44] reported the beneficial effects in the lipid profile of Sprague Dawley rats with hepatic fibrosis induced with CCl_4_ treated with yam. Its administration decreased serum triglyceride and LDL-c levels although no effects were found in serum cholesterol concentration.

## 5. Conclusion 

The tamarind tablets inhibit lipid peroxidation in Sprague Dawley rats intoxicated with CCl_4_. The redox balance (SOD-CAT-GSH) was normal in the experimental groups treated with tablets in both dosage levels. There are no statistical significant differences among the results observed for Silymarin 150 mg/kg and the tamarind tablets at both dose levels. The Tamarind tablets keep the liver functions in the presence of the CCl_4_ at levels that were not statistically different from the values observed in the control group. The dose 200 mg/kg of tablets was the best, because it kept liver function in the experimental animals. These results justify, scientifically, the ethnobotanical use of the leaves of* Tamarindus indica* L.

## Figures and Tables

**Table 1:** Corporal weight gain and relation between rat liver and final corporal weight (standard deviation on parenthesis).

Group	Initial body weight (g)	Final body weight (g)	Body weight gain (g)	Liver weight (g)	Liver weight/final body weight (%)
I	176.39 (12.23)	212.23 (13.42)	35.84 (3.25)^b^	3.49 (0.80)^a^	1.64 (0.22)^a^
II	189.24 (13.88)	194.70 (17.40)	5.46 (1.99)^a^	4.85 (0.86)^b^	2.49 (0.51)^b^
III	175.29 (12.26)	214.87 (25.21)	39.58 (4.52)^b^	3.41 (0.75)^a^	1.59 (0.29)^a^
IV	181.36 (12.54)	223.47 (13.15)	42.11 (4.12)^b.c^	3.86 (0.65)^a,b^	1.72 (0.35)^a^
V	191.86 (13.47)	229.93 (16.69)	38.07 (3.57)^b^	3.75 (0.92)^a,b^	1.63 (0.26)^a^

I: control group; II: CCl_4_ induced hepatotoxicity group; III: Silymarin treated group; IV and V: *Tamarindus indica* L. tablets treated groups at doses 100 mg/kg and 200 mg/kg, respectively.

Different letters on columns indicate significant statistical differences (*p* < 0.05).

**Table 2:** Antioxidant defense system in the liver of Sprague Dawley rats with hepatotoxicity induced by CCl_4_ treated with *Tamarindus indica* L. tablets.

Group	MDA	SOD	CAT	GSH
	(nmol/g tissue)	(UI/mg tissue)	(UI/mg tissue)	(mg/g tissue)
I	0.47 ± 0.04^c^	0.35 ± 0.02^a^	0.40 ± 0.02^a.b^	29.74 ± 1.98^b^
II	1.49 ± 0.18^a^	0.15 ± 0.01^d^	0.13 ± 0.02^d^	15.84 ± 1.30^c^
III	0.66 ± 0.03^b^	0.31 ± 0.02^b.c^	0.36 ± 0.02^c^	32.58 ± 2.68^a.b^
IV	0.60 ± 0.04^b.c^	0.28 ± 0.03^c^	0.43 ± 0.02^a^	33.01 ± 2.26^a^
V	0.64 ± 0.03^b^	0.33 ± 0.02^a.b^	0.37 ± 0.03^b.c^	29.93 ± 0.90^b^

MDA: malondialdehyde; SOD: superoxide dismutase; CAT: catalase; GSH: reduced glutathione.

I: control group; II: CCl_4_ induced hepatotoxicity group; III: Silymarin treated group; IV and V: *Tamarindus indica* L. tablets treated groups at doses 100 mg/kg and 200 mg/kg, respectively.

Data are expressed as mean ± SD (*n* = 7).

Different letters on columns indicate significant statistical differences (*p* < 0.05).

**Table 3:** Effect of *Tamarindus indica* L. tablets on serum biochemical markers of liver function in CCl_4_ induced hepatotoxicity in Sprague Dawley rats.

Group	ALT (UI/L)	AST (UI/L)	ALP (UI/L)	GGT (UI/L)	TB (mg/100 mL)	TP (g/100 mL)
I	32.56 ± 2.05^b^	21.37 ± 0.59^c^	187.32 ± 3.08^d^	86.18 ± 3.86^d^	3.09 ± 0.04^d^	7.12 ± 0.47^b^
II	326.78 ± 19.31^a^	95.97 ± 4.41^a^	426.53 ± 3.31^a^	161.30 ± 7.90^a^	6.57 ± 0.09^a^	5.26 ± 0.37^c^
III	36.33 ± 0.94^b^	37.27 ± 5.64^b^	204.56 ± 2.90^c^	98.10 ± 1.40^c^	4.84 ± 0.25^c^	8.29 ± 0.22^a^
IV	38.62 ± 0.98^b^	38.67 ± 2.50^b^	246.76 ± 3.10^b^	111.10 ± 1.19^b^	5.21 ± 0.17^b^	7.41 ± 0.23^b^
V	33.49 ± 0.91^b^	25.38 ± 2.38^c^	202.51 ± 4.96^c^	99.09 ± 3.34^c^	5.15 ± 0.06^b^	8.33 ± 0.34^a^

ALT: alanine aminotransferase, AST: aspartate aminotransferase, ALP: alkaline phosphatase, GGT: *γ*-glutamyl transpeptidase, TB: total bilirubin, and TP: total protein.

I: control group; II: CCl_4_ induced hepatotoxicity group; III: Silymarin treated group; IV and V: *Tamarindus indica* L. tablets treated groups at doses 100 mg/kg and 200 mg/kg, respectively.

Different letters on columns indicate significant statistical differences in Tukey HSD test.

Data are expressed as mean ± SD; *n* = 7.

**Table 4:** Effect of *Tamarindus indica* L. tablets on serum lipid profile in CCl_4_ induced hepatotoxicity in Sprague Dawley rats.

Group	TG (mg/dL)	CHOL (mg/dL)	HDL-c (mg/dL)	LDL-c (mg/dL)	VLDL-c (mg/dL)
I	48.41 ± 2.26^c^	94.91 ± 3.16^c^	49.59 ± 1.83^a^	35.64 ± 1.75^b^	9.68 ± 0.95^b^
II	120.57 ± 6.90^a^	151.00 ± 3.43^a^	25.98 ± 2.06^c^	100.91 ± 5.43^a^	24.11 ± 1.32^a^
III	56.88 ± 2.94^b^	96.56 ± 2.78^b^	51.24 ± 1.16^a^	33.94 ± 2.02^b^	11.38 ± 1.59^b^
IV	52.32 ± 3.39^c^	92.58 ± 2.99^c^	46.41 ± 1.40^b^	35.71 ± 0.98^b^	10.46 ± 0.98^b^
V	50.83 ± 2.04^c^	90.59 ± 2.71^c^	48.29 ± 1.33^b^	32.13 ± 1.95^c^	10.17 ± 0.91^b^

TG: triglycerides, CHOL: cholesterol, HDL: high-density lipoproteins, LDL: low-density lipoproteins, and VLDL: very low-density lipoproteins (-c, bounded to cholesterol).

I: normal group; II: CCl_4_ induced hepatotoxicity group; III: Silymarin treated group; IV and V: *Tamarindus indica* L. tablets treated groups at doses 100 mg/kg and 200 mg/kg, respectively.

Different letters on columns indicate significant statistical differences in Tukey HSD test.

Data are expressed as mean ± SD; *n* = 7.
